# Immune inhibitory receptor agonist therapeutics

**DOI:** 10.3389/fimmu.2025.1566869

**Published:** 2025-03-26

**Authors:** Rustin R. Lovewell, Solomon Langermann, Dallas B. Flies

**Affiliations:** NextCure, Beltsville, MD, United States

**Keywords:** immune, inhibitory, agonist, therapeutic, receptor, SuSu, SuSt

## Abstract

The immune system maintains the health of an organism through complex sensing and communication mechanisms. Receptors on the surface of immune cells respond to stimuli resulting in activity described at its most basic as inhibitory or stimulatory. Significant progress in therapeutic intervention has occurred by modulating these pathways, yet much remains to be accomplished. Therapeutics that antagonize, or block, immune inhibitory receptor (IIR) pathways, such as checkpoint inhibitors in cancer are a key example. Antagonism of immune stimulatory receptors (ISRs) for dysregulated inflammation and autoimmunity have received significant attention. An alternative strategy is to agonize, or induce signaling, in immune pathways to treat disease. Agonism of ISRs has been employed with some success in disease settings, but agonist therapeutics of IIRs have great, untapped potential. This review discusses and highlights recent advances in pre-clinical and clinical therapeutics designed to agonize IIR pathways to treat diseases. In addition, an understanding of IIR agonists based on activity at a cellular level as either agonist suppression of stimulatory cells (SuSt), or a new concept, agonist suppression of suppressive cells (SuSu) is proposed.

## Introduction

Plasma cell membrane molecules known broadly as cell surface receptors provide cells, especially immune cells, with the ability to sense and communicate with the external environment (1). This includes cell-to-cell communication by interaction with ligands expressed on other cells, as well as the ability to sense and respond to non-cellular ligands, including pathogens, soluble factors, and extracellular matrix molecules (2). Cell surface receptors link to cytoplasmic molecules that engage in ordered interactions resulting in signal propagation (1, 2). Integration of these signaling pathways direct immune cell activity.

**Figure 1:**
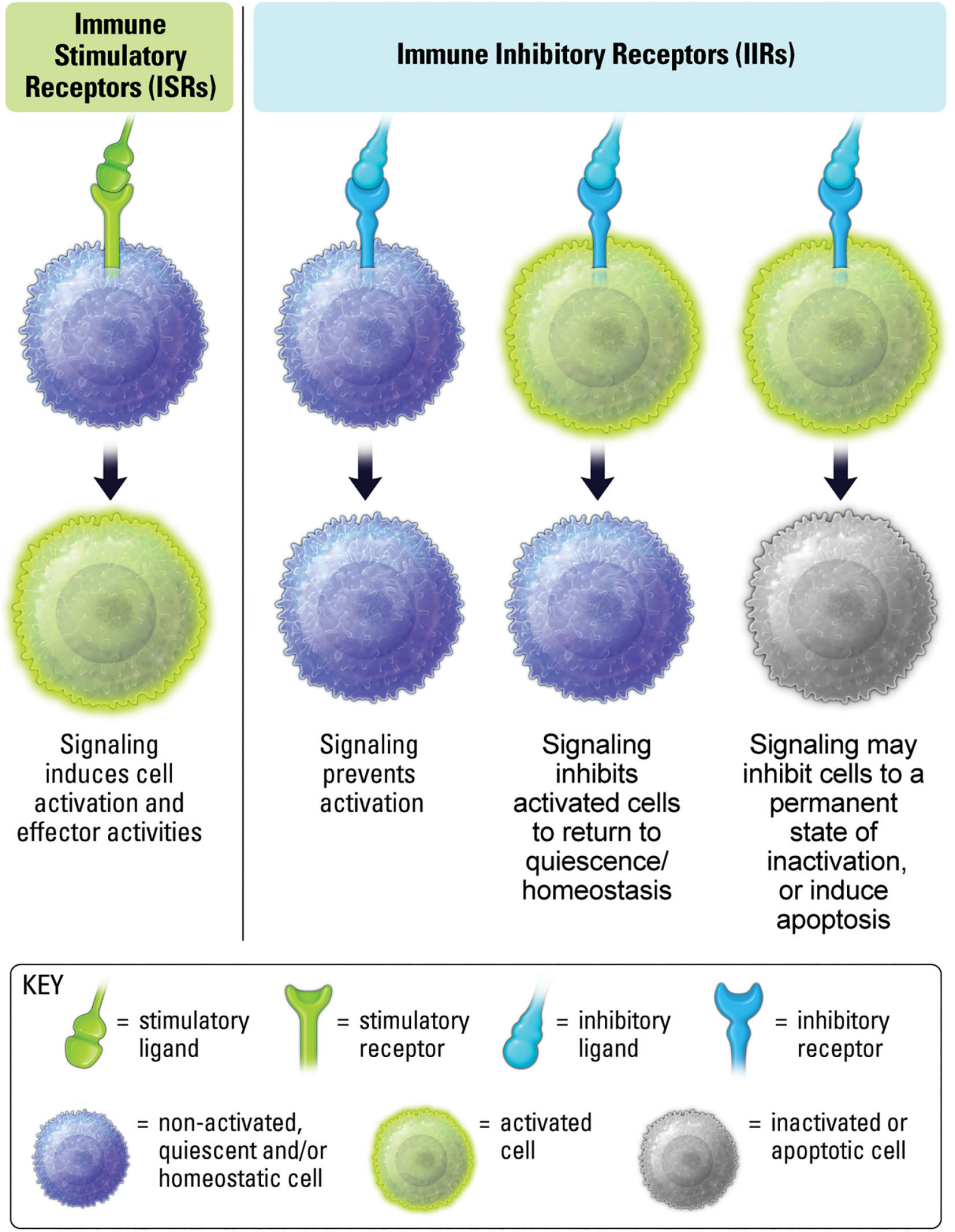
ISRs interact with ligands leading to activation and effector activity. IIR signaling prevents stimulation, inhibits previously stimulated cells, and may induce inactivity and cell death.

Immune stimulatory and inhibitory receptors, ISRs and IIRs, respectively, encompass a broad range of immune cell surface receptors that function to regulate the duration and magnitude of immune responses (1, 3). As the name implies, ISRs induce signaling pathways that increase immune activity, generally through kinase-mediated phosphorylation of proteins that induce structural changes in molecules resulting in signal propagation (**Figure 1**) (4). IIRs down-modulate signal propagation, and counter-act ISR pathways, generally through phosphatase-mediated de-phosphorylation of activation pathways (**Figure 1**) (5, 6). IIRs can also maintain self-tolerance, quiescence, and homeostasis in inflammatory settings (7, 8).

## Immune regulation by IIRs

The human genome may encode over 300 IIRs (3, 7). IIRs are often considered receptors that dampen T cell receptor (TCR) signaling and the propagation of T cell responses (9, 10). An expanded definition encompasses immunomodulatory receptors that broadly regulate both adaptive and innate cell processes (11–13). Recognition of this broader repertoire of IIRs, and the corresponding downstream signaling pathways, has expanded biological understanding of immune regulation. However, the majority of IIRs are poorly characterized, so much work is needed to understand the nuances of IIR biology.

**Figure 2:**
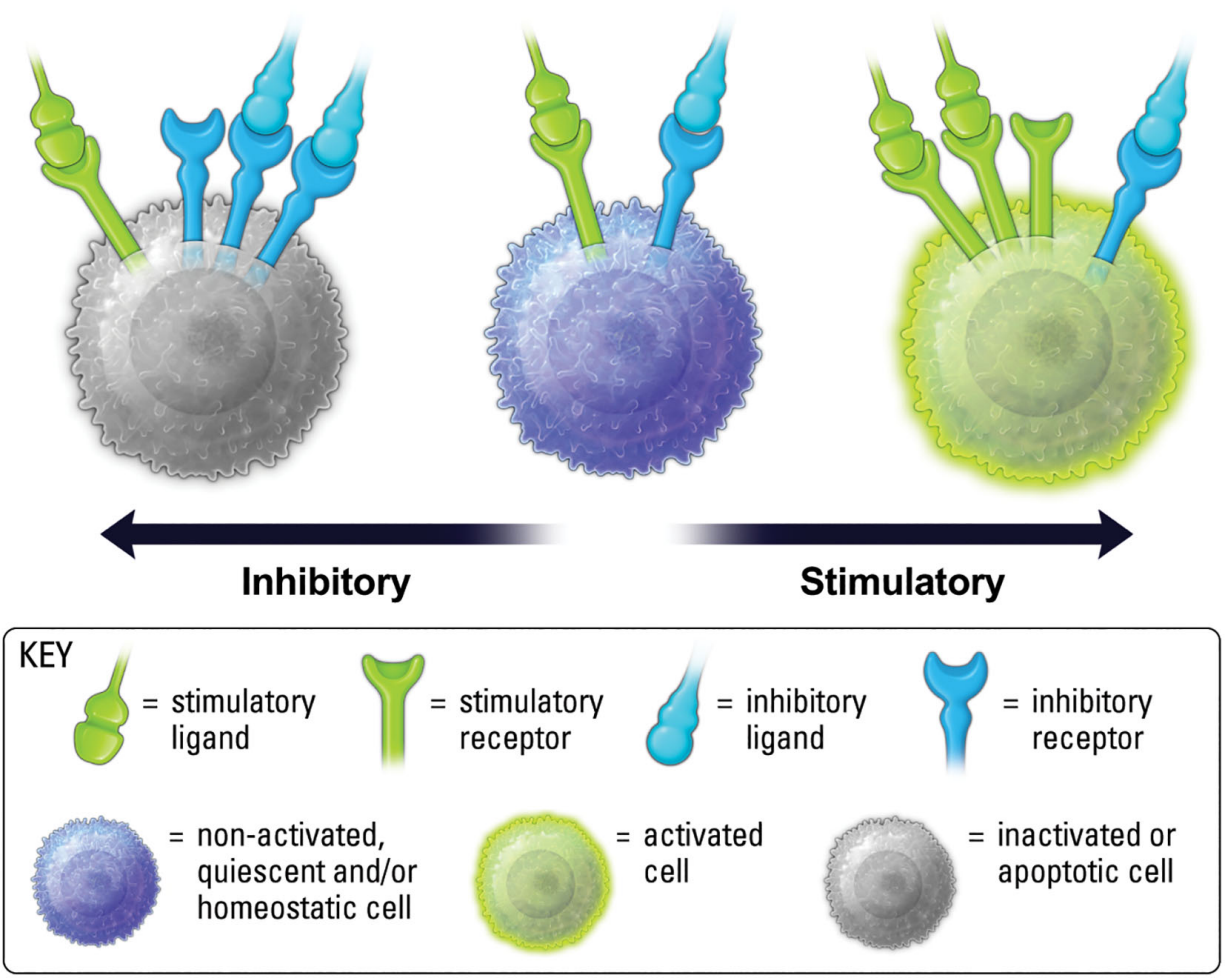
Balance of ISR and IIR levels on the cell surface, along with ligand availability and binding kinetics, are important for determining cell phenotype.

The balance of stimulatory versus inhibitory signals is a vital determinant of immune cell phenotype and function (**Figure 2**) (14). Studies on IIR biology have revealed the critical role for inhibitory signaling in maintaining homeostasis in the presence of stimuli (14–17). Loss of function mutations in IIR pathways are responsible for many chronic inflammatory disorders (8, 18, 19), and one of the hallmark adversities facing approved immune checkpoint inhibitors (CPIs) is the incidence of autoreactive T cells that arise from persistent blockade of IIR signaling (20, 21). As such, when IIR expression levels or signaling capability is decreased, stimulatory activity may increase in contextual settings, such as inflammation, where IIRs are needed to maintain homeostasis.

## Agonist versus antagonist therapeutics

Therapeutics are characterized by their functional effect on a cognate antigen (22). A therapeutic targeting the same antigen can have very different effects depending primarily on the epitope, the part of the antigen/molecule to which the therapeutic binds, as well as other factors of the therapeutic reviewed for mAbs in (22).

A therapeutic described as an antagonist, for the purposes of this review, is a drug that binds to a molecular epitope on either a receptor or a ligand that disrupts (blocks) the function of a natural receptor-ligand interaction. While the natural function of a receptor-ligand interaction may be other than cell signaling, such as adhesion, simply put for the purpose of this review, an antagonist will bind to a molecular epitope that disrupts or blocks the signaling capability of a receptor.

Conversely, for purposes of this review, an agonist therapeutic will engage a molecular epitope on a receptor that induces downstream, intracellular signaling pathways that confer functional outputs in a cell. Agonists bind to epitopes that mimic, enhance, or add to a natural, cognate receptor-ligand interaction resulting in cell signaling. As such, an agonist therapeutic may bind the identical epitope as a natural ligand, or a different epitope than a natural ligand(s). This means an agonist may have the additional function of blocking the natural receptor-ligand interaction, or if the therapeutic binds to a different epitope than the natural ligand, the agonist therapeutic may synergize with natural ligands for enhanced signaling. These points will be further touched upon in this review, but details can be reviewed elsewhere (22).

**Figure 3:**
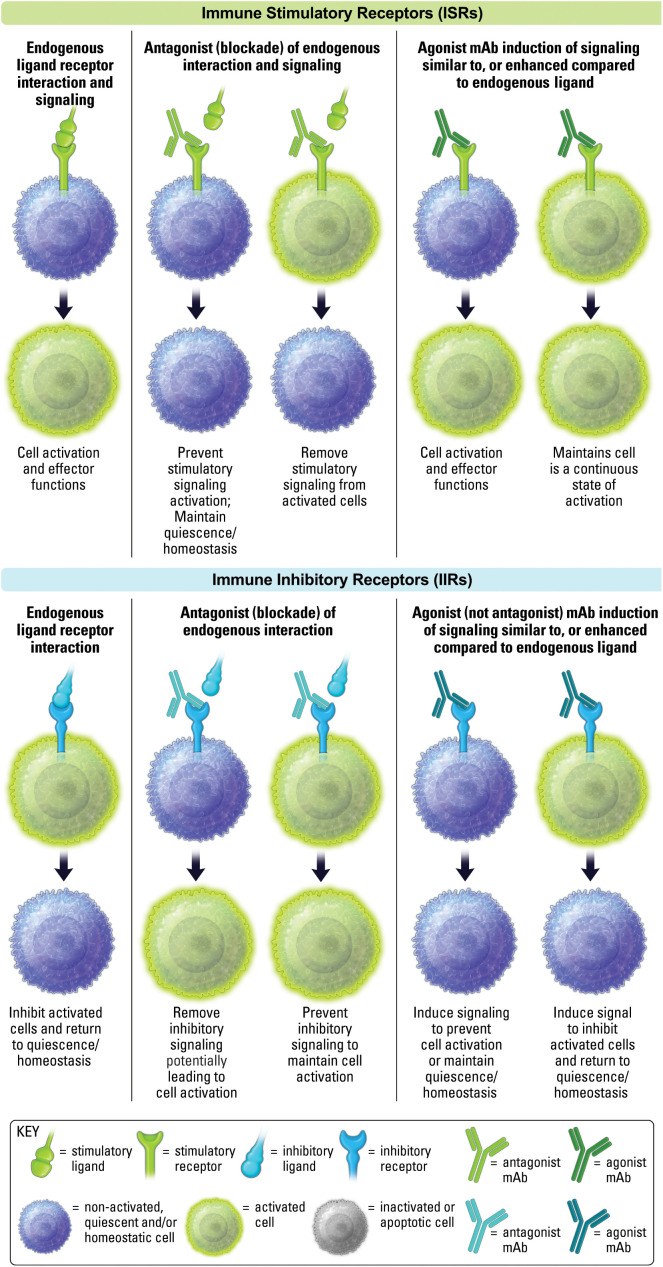
Antagonism and agonism of ISRs and IIRs. The effect of endogenous ligand is shown on the left. The middle depicts antagonist activity of non-activated and activated cells. The right side depicts agonist activity of non-activated and activated cells.

From a therapeutic perspective, antagonism (blockade) of IIRs has been the primary approach of cancer immunotherapy, and are usually referred to as checkpoint inhibitors (CPIs), with PD-1 being the quintessential example. Antagonism of ISRs to block stimulatory receptor signaling is an important approach for autoimmune and inflammation-associated diseases, with CTLA4-Ig, otherwise known as Abatacept, being a quintessential example of blockade of CD28 ISR receptor interaction with both B7-1 and B7-2 ligands (23). Antagonist therapeutics will bind target receptors or ligands and obstruct and/or outcompete natural receptor-ligand interactions, thereby preventing receptor-mediated signaling (**Figure 3**). CPIs that block IIRs to promote immune responses continue to yield clinical success for many cancer patients (24), as does the blockade of ISR pathways in patients with autoimmune or inflammatory disease, or those undergoing transplantation (25–27).

As mentioned above, an agonist therapeutic will promote signal transduction through a receptor that generally mimics a natural endogenous ligand, but may also enhance signaling in comparison to endogenous ligand (**Figure 3**). Agonists against ISRs have been a focus of attention in cancer. While an early CD28 super agonist, TGN1412, famously failed years ago, 4-1BB (CD137), OX40, GITR, CD40, new CD28 agonists and other ISR agonists have continued to make progress as therapeutics (28, 29). ISR agonists will not be discussed in detail in this review and can be review elsewhere (30, 31).

**Figure 4:**
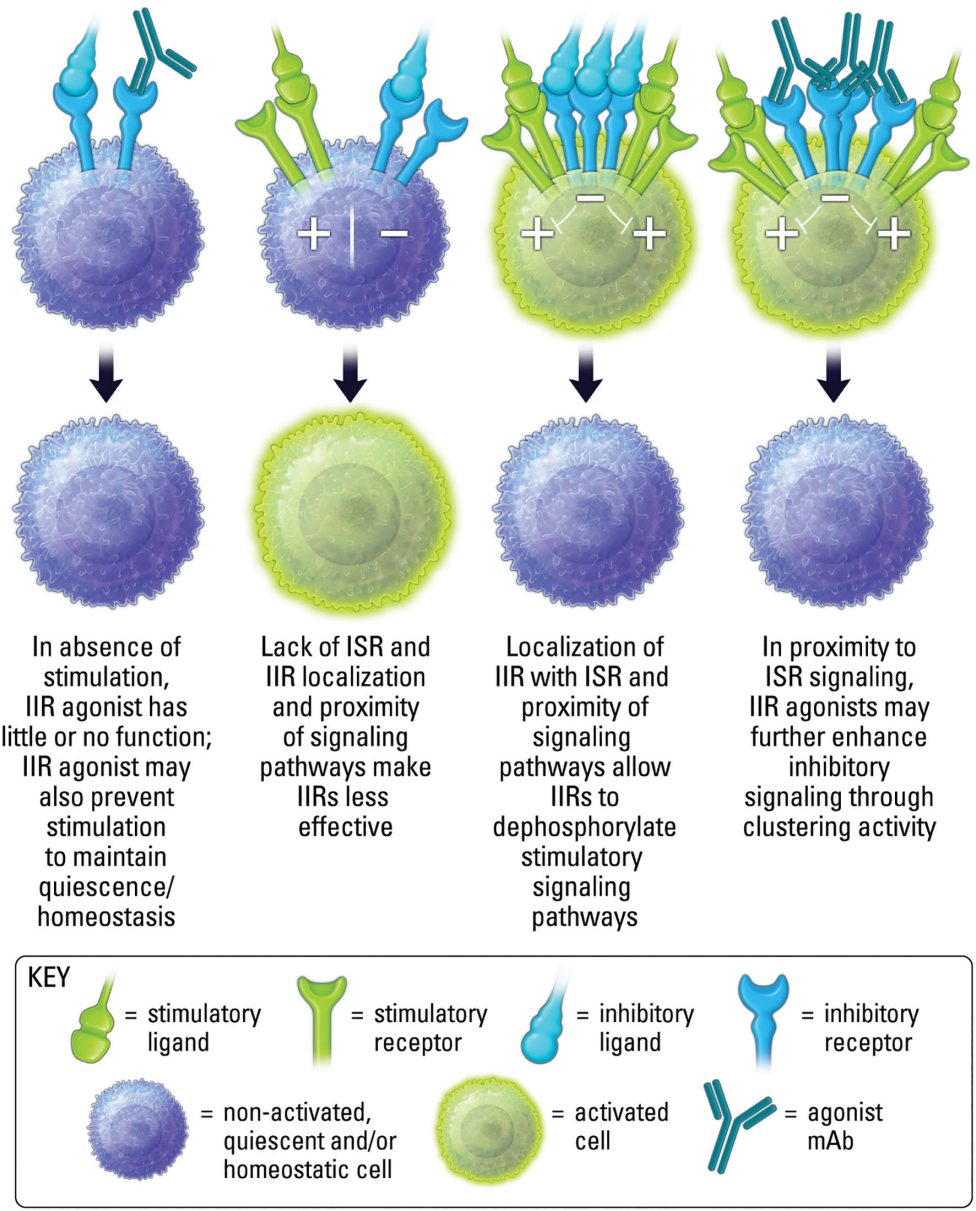
Localization, proximity and activation state are important for both ligand induced inhibitory receptor function, and agonist therapeutic activity.

**Figure 5:**
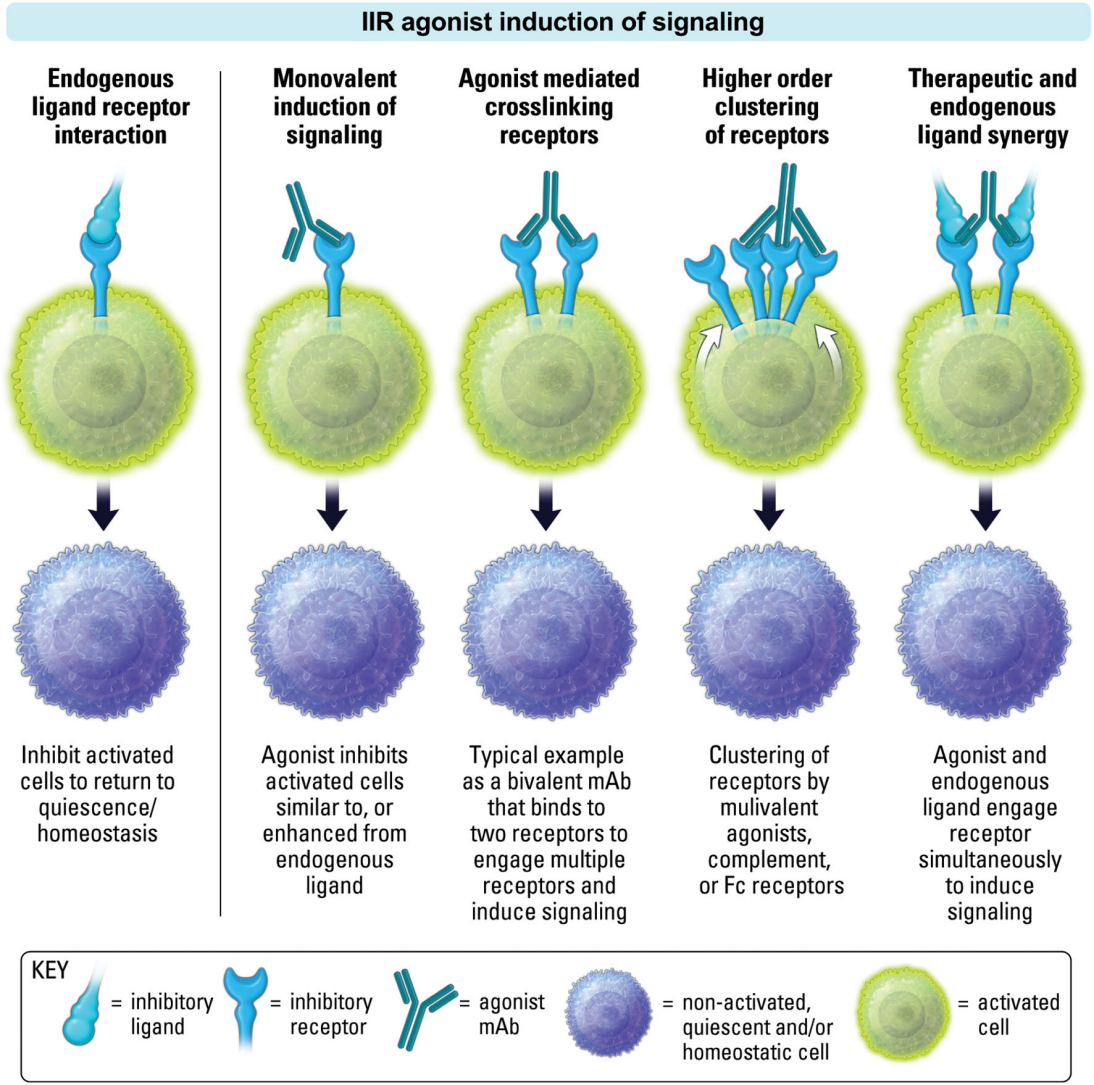
IIR agonist induction of signaling is dependent on both the receptor ability to signal in monovalent or multivalent fashion, and the agonist ability to crosslink or cluster receptors. An IIR agonist may synergize with endogenous ligand as shown on the right.

The clinical path for IIR agonist therapeutics, in comparison to ISR therapeutics, has received less attention until recently. This is partly due to limitations in clearly understanding the characteristics that make a good agonist. Of key importance is the spatial requirement of IIRs (**Figure 4**). Receptor localization often dictates function (18). This means the spatial proximity of a sufficient number of IIRs can also dictate function. As such, an agonist often requires receptor clustering or crosslinking. In the case of an antibody, this may occur via the antibody’s multivalent binding regions (**Figure 5**). Oftentimes clustering and crosslinking by mAbs requires the IgG Fc region of a mAb to bind to Fc receptors expressed on immune cell populations. As such, many agonist mAbs are engineered with IgG Fc regions designed to enhance their engagement with Fc receptors (FcR) as a key mechanism of increasing crosslinking and clustering of IIRs (and ISRs) (32).

Importantly, the proximity of IIR and ISR receptors, and the respective signaling components, is crucial to the ability of IIR-mediated regulation of ISR stimulatory pathways (**Figure 4**) (33, 34). Therefore, in conditions lacking proper stimuli, and where receptor localization is absent, the activation of phosphatases by an IIR agonist may be limited (**Figure 4**). In addition, IIR signaling in the absence of localization with ISRs may be inconsequential, since phosphatases may have less effect when spatially distanced from stimulatory components (**Figure 4**) (7, 14). Moreover, as mentioned above, it is important to recognize that while an agonist will most often block endogenous ligand binding to a cognate receptor due to a shared binding epitope, in some cases synergy may occur between agonist and endogenous ligand if both are capable of binding a receptor simultaneously. Finally, because IIR agonism is inducing signaling, it should be considered an active process of inhibiting cell activity, rather than the passive method of removing (blocking) a stimulatory signal to decrease cell activity, as occurs with antagonism if ISRs. As the repertoire of clinical stage immunotherapeutics targeting IIRs grows, recognizing mechanistic differences will be important for designing effective clinical strategies. The majority of this review will focus on mAb IIR agonists, but we will also touch upon emerging small molecule IIR agonists.

## IIR agonism at a cellular level: SuSt or SuSu

**Figure 6:**
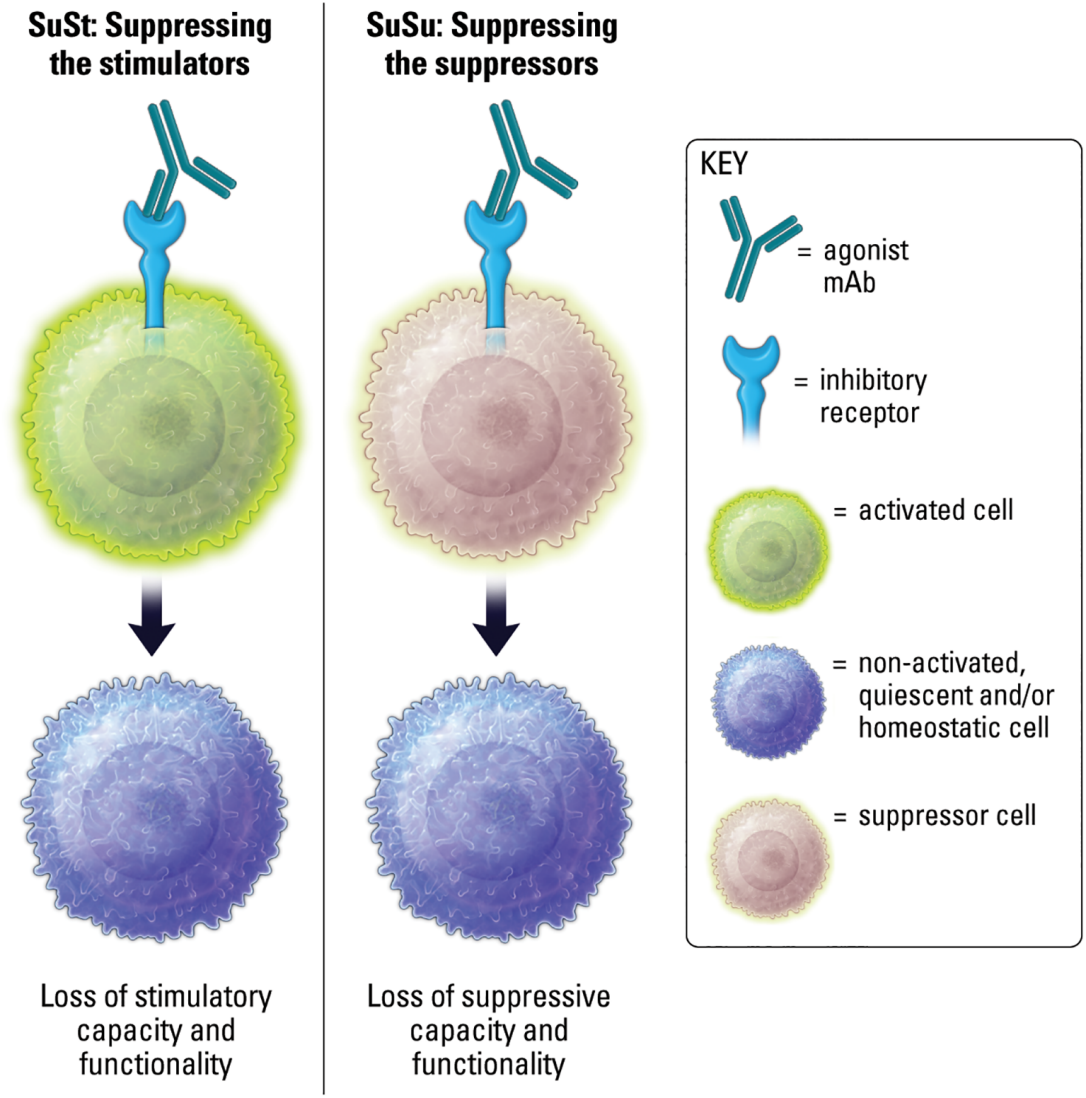
Agonism of IIRs on stimulated or stimulatory cells results in loss of stimulatory capacity and functionality. Agonism of IIRs on suppressor cells may result in loss of suppressive capacity and functionality.

The ultimate functional outcome of targeting cell surface immune receptors is to effect change on a cellular level (15). The functional outcome of IIR agonists on cellular activity is context-dependent, as is generally the case with immunity, and generally results in two over-arching outcomes. The first is the well-understood notion of inducing IIR signaling to suppress stimulated or stimulatory cell functions (8). These may be called “suppress the stimulator” (SuSt) agonists (**Figure 6**). The second outcome is a more counter-intuitive and speculative concept of suppressing the suppressive cells. These therapeutics may be called “suppress the suppressor” (SuSu) agonists (**Figure 6**). SuSt agonists are commonly utilized in autoimmune and inflammatory diseases. Whereas a more novel concept can be considered for SuSu agonists in contextual conditions, such as cancer, where suppressive cell populations, including myeloid derived suppressor cells (MDSCs) and regulatory T cells (Tregs), can actively suppress beneficial anti-tumor immune responses, and where IIR SuSu agonists could inhibit key functions of these suppressor cells to restore proper immunity.

It should be noted that the concept of induced suppression of suppressive cell mechanisms of action by IIR agonism would be considered an active process, altogether different from blocking inhibitory receptors on suppressive cells, which in simple terms is thought to convert a suppressive cell phenotype into a less suppressive or stimulatory phenotype. As an example, targeting a tumor-associated macrophage (TAM) with an IIR antagonist blocking mAb may promote conversion toward an “M1” like-state and generate the associated stimulatory cytokines (35). However, a SuSu agonist might shut down TAM trafficking, production of inhibitory cytokines, and potentially induce apoptosis, rather than converting or reverting the TAM into a different cell phenotype.

## Preclinical and clinical IIR agonist therapeutics

Therapeutic modalities continue to expand, but here we focus on two major categories of biologics and small molecules. Agonist monoclonal antibodies (mAbs) against ISRs such as CD28, 4-1BB, CD40, and other Tumor Necrosis Family Receptors (TNFRs) have been in clinical testing for cancer for many years and are reviewed elsewhere (31). Agonist monoclonal mAbs or proteins against IIRs are now emerging for intervention against inflammatory and autoimmune disorders (8, 18). Small molecule agonism of IIRs is also advancing concurrent with progress in the biologics space. The following sections describing research and clinical development of IIR agonists is broken down into three sections. The first is biologic agonists in autoimmune and inflammation-associated diseases. The second section is on small molecule agonists in autoimmune and inflammation-associated diseases. The third section is the potential use of IIR agonists in cancer.

## Biologic IIR agonists in inflammation and autoimmune disease

The past few decades have seen important advances in understanding of the biology of IIRs. This knowledge is now being leveraged toward novel agonist treatments for multiple non-oncology disorders (**Table 1**) (18, 36). From a cellular, functional outcome point-of-view, agonist targeting in the inflammation and autoimmune disease setting are routinely considered SuSt type agonist therapeutics designed to suppress aberrantly stimulatory or activated cell populations.

**Table 1:** IIR targets and agonists therapeutics.

Target	Agonist	Company	Indication	Highest Stage Achieved
PD-1	Peresolimab	Eli Lilly	Rheumatoid Arthritis	Phase II
	Rosnilimab (ANB030)	Anaptysbio	Rheumatoid arthritis and Ulcerative Colitis	Phase II
	CC-90006	Anaptysbio/ Bristol Myers Squibb (ex Celgene)	Psoriasis	Phase II
	PT627	Merck & Co (ex Pandion Therapeutics)		Preclinical
	MB151	Gilead (ex Mirobio)		Preclinical
	RTX-002	Ibio (ex RubrYc)		Preclinical
LAG-3	IMP761	Immutep		Phase I
BTLA	BTLA	AnaptysBio	Atopic Dermatitis	Phase II
ChemR23	OSE-230	OSE Immunotherapeutics	Inflammatory Bowel Disease	Preclinical
SIRPα	name undisclosed	Genentech	Rheumatoid Arthritis and Inflammatory Bowel Bisease	Preclinical
PSGL-1	Neihulizumab	Altrubio	GvHD, Psocriatic Arthritis, Ulcerative olitis	Phase II
LAIR-1	NC525	NextCure	AML, CMML, MDS	Phase I
CD200R	LY345738/Ucenprubart	Eli Lilly	Atopic Dermatitis	Phase II
VSTM-1	Name undisclosed	NextCure	COPD, Inflammatory Bowel Disease	Preclinical
VISTA	INX803	Immunext		Preclinical
	7G1 (37)	Sunnybrook Research Institute/University of Toronto		Preclinical
	M351-0056, Imatinib, Baloxavir marboxil	Nanjing University	Autoimmune Disease	Preclinical

### PD-1/PD-L1

PD-1 remains the most clinically targeted immune checkpoint molecule across all indications (38). PD-1 is expressed on T cells, is associated with T cells with reduced anti-tumor function, and promotes inhibitory signaling when engaged to PD-L1, which is expressed on tumor cells and some immune cell subpopulations (1). Antagonism of the PD-1/PD-L1 interaction has proven successful in treating cancer. Currently, the inverse strategy of agonizing PD-1 to dampen hyperreactive T cells in certain autoimmune/inflammatory conditions has been gaining momentum (39–44). As mentioned above, an antagonist vs agonist therapeutic depends on the epitope of PD-1 to which the therapeutic binds. It is beyond the scope of this review to discuss in detail specific epitopes of agonists and antagonists, to the extent this is known and in the public domain, but some detail can be found here (22, 45). While the PD-1 agonist mAb CC-90006 has been tested in psoriasis patients since 2016 (NCT03337022), the most clinically advanced PD-1 agonist to date is peresolimab (LY3462817), currently in a phase II clinical trial for patients with moderately-to-severely active rheumatoid arthritis (NCT05516758). Similarly, Luu et al. recently reported that the PD-1 agonist rosnilimab reduced peripheral T cell proliferation, cytokine secretion, and circulating PD-1^High^ T cells in a Phase 1 safety and tolerability trial (NCT06127043), showcasing the overall potential of anti-PD-1 agonist therapeutics.

### VISTA/PD-1H

V-domain immunoglobulin suppressor of T cell activation (VISTA), also called Programmed Death-1 Homolog (PD-1H), is a type I transmembrane inhibitory receptor with expression restricted to immune cells (46–49). VISTA is expressed on both T cell and myeloid cell compartments and maintains immune cell quiescence (50). VISTA was first identified as an inhibitory receptor expressed on T cells (PD-1H). An important difference between PD-1 and PD-1H is that PD-1 is expressed on activated T cells, while PD-1H can be expressed on naïve T cells and regulatory T cells, as well post-priming T cells (46–48). As such, while agonist targeting of PD-1 may target resolution of disease, PD-1H/VISTA agonist target may potentially prevent, reduce and resolve disease.

To date, human agonist mAbs against VISTA have been challenging to identify, and no agonist mAbs against VISTA have been employed in the clinic for treatment of inflammatory disease, but strong preclinical findings in murine studies have highlighted the potential for clinical development (51). Specifically, VISTA agonist antibodies suppressed alloreactive T cells in mouse models of GvHD, acute inflammation, and acute hepatitis (46–48). Likewise, suppressed autoimmunity was observed in models of systemic and cutaneous lupus erythematosus (52), and reduced lung inflammation and disease severity was observed in experimental asthma models (53). These findings are further supported by studies showing that mice treated with VISTA agonists displayed decreased nuclear factor-κB (NF-κB) signaling, increased survival, and improved disease score in models of hepatitis, arthritis, and psoriasis (54), as well as decreased pulmonary fibrosis during bleomycin-induced fibrotic disease (55). Interestingly, a group has identified at least two FDA-approved small molecules as agonists of VISTA (further discussed below), which may provide a viable alternative to developing agonist mAbs (56, 57).

### PSGL-1

P-selectin glycoprotein ligand-1 (PSGL-1, CD162), while not necessarily considered an inhibitory receptor, but rather an adhesion molecule that binds to P selectin to regulate T cell migration and function (58), also has inhibitory signaling activity. Moreover, PSGL-1 has been shown to bind to the inhibitory molecule VISTA, particularly under acidic conditions in tumor microenvironments, where this interaction appears to play a role in suppressing anti-tumor immunity (59). Preclinical models of GvHD and type I diabetes demonstrated that an agonist mAb against PSGL-1 could promote apoptosis of hyperactivated T cells without affecting the binding of P selectin (60). These data led to the development of neihulizumab (ALTB-168) and a phase II clinical trial in patients with psoriatic arthritis and ulcerative colitis (NCT03298022). The trial showed promising clinical responses but unfortunately did not reach completion due to site operational difficulties associated with the COVID-19 pandemic.

Multivalent antibodies with >2 antigen binding regions have been shown to form higher-order receptor super-clusters compared to bivalent counterparts, which can in turn drive better agonist activity (61–63). The second-generation follow-up to neihulizumab, leiolizumab (ALTB-268), is a tetravalent molecule with 4 antigen binding domains which promote receptor super-clustering and thus improve activity over the parent molecule, and currently in a phase II study in subjects with moderately-to-severely active ulcerative colitis (NCT06109441).

### LAIR-1

Leukocyte Associated Immunoglobulin Like Receptor 1 (LAIR-1, CD305) is a hematopoietic cell restricted IIR that binds to collagen-domain containing ligands to dampen cellular responses (64). The anti-inflammatory role of LAIR-1 has been well-documented, yet LAIR-1 has differential functions depending on the disease setting (16, 65–67). LAIR-1 has similarities to VISTA/PD-1H in expression pattern; it is expressed on both naïve and post-priming T cells, and is broadly expressed on other immune populations as well (7, 68). With this in mind, agonists targeting LAIR-1, as with VISTA, could help to prevent, reduce and resolve disease, rather than only targeting the resolution phase of disease. A LAIR-1 agonist IgG1 mAb was developed for the treatment of heme cancers (clinical trial NCT05787496) based on its role in promoting leukemic cell apoptosis (67, 69). However, the ligation of LAIR-1 by agonist mAbs can also inhibit myeloid cell Toll-like receptor 4 (TLR-4) and Interferon alpha (IFN-α) induced signaling, phenotypic differentiation, and cytokine expression, as well as NK cell- mediated cytolysis and B cell receptor-mediated signaling (16, 70, 71). As such, LAIR-1 agonists are a highly promising treatment for patients with inflammatory and autoimmune disease.

### TIGIT

T cell immunoreceptor with Ig and ITIM domains (TIGIT, VSIG9, VSTM3, WUCAM) is another well-described IIR targeted in advanced clinical trials, often targeted with blocking antibodies to restore T cell function in cancer (72). Joller, Kuchroo and colleagues identified TIGIT and first identified both agonist and antagonist antibodies (73). While specific mAb clones were identified with either antagonist, agonist, or both properties, that bound to different epitopes of TIGIT, the specific binding site of these mAbs was not elaborated on (74). An agonist antibody decreased effector T cell proliferation and cytokine production, but also enhanced IL-10 transcription in Tregs. TIGIT signaling may reduce protein kinase B (Akt)/mammalian target of rapamycin (mTOR) activity and signal transducer and activator of transcription 4 (STAT4) signaling, and can also act upstream of TCR signaling to reduce expression of the TCR signaling complex (75). Because of the multiple effects of TIGIT on effector T cells and Tregs, agonist targeting of TIGIT appears promising. Studies continue to emerge linking the expression of TIGIT to autoimmune disorders, as well as the benefit of agonist targeting of TIGIT in settings such as systemic lupus erythematosus (SLE), Sjogren’s syndrome, and transplantation (76–78). In SLE, specifically, agonism of TIGIT by the ligand protein CD155 delayed progression of disease in a mouse model of SLE through down-modulation of CD4+ T cells (79). In addition, engineering a PD-1/TIGIT dual activating nanoparticle with dexamethasone was demonstrated to act synergistically to treat SLE (80).

### LAG-3

Lymphocyte-activating gene-3 (LAG-3, CD223) is an IIR and well-established checkpoint that has also been broadly targeted for cancer immunotherapy. Relatlimab (LAG-3 blocking antibody) in combination with nivolumab has been approved for patients with advanced melanoma (81). A LAG-3 agonist antibody IMP761 has been shown to inhibit TCR-mediated nuclear factor of activated T cells (NFAT) activation, antigen-induced human T cell proliferation, and, in a cynomolgus macaque model of antigen-specific delayed-type hypersensitivity (DTH), the antibody suppressed pathogenic T cell responses (82). IMP761 has progressed to a Phase I dose escalation study in healthy volunteers (NCT06637865). It is likely that additional innovative and engineering strategies will emerge to agonize LAG-3 for the treatment of autoimmune disease.

### BTLA

BTLA is a T and B cell-expressed IIR that is upregulated in rheumatoid arthritis, and variable expression and/or signaling capacity is several other autoimmune diseases (83). ANB032 is a BTLA agonist mAb was in Phase II clinical trials for treatment of atopic dermatitis (AD) (NCT05935085). In addition to being expressed on B and T cells, BTLA is also expressed on Dendritic Cells (DCs). A primary mechanism of ANB032 is agonist inhibition of DC maturation, thus reducing antigen presentation as well as ISR ligand expression (84). In addition, ANB032 has also been shown to induce regulatory T cells, but not effector T cells, thus reducing multiple T cell produced cytokines associated with autoimmunity and inflammation. The mAb contains an IgG4 Fc region engineered to enhance FcR engagement and promote BTLA signal transduction (32, 84). Although ANB032 did not meet primary or secondary endpoints in AD and the trial was ended, the agonist mAb was well tolerated and safe. While the Phase II trial treated patients that were anti-IL-13 or dupilmab naïve or experienced, it is interesting to speculate if ANB032 would have benefit in combination settings.

### CD200R

CD200R is an inhibitory receptor expressed on several immune subsets including macrophages, DCs, activated T cell subsets and mast cells (85). Humans appear to have only one functional isoform, CD200R1, while rodents may have additional splice variants (85). Upon CD200R binding to CD200 ligand expressed on both immune and non-hematopoietic cells, two (human) cytoplasmic domain tyrosine residues are phosphorylated and recruit inhibitory adaptor proteins Dok1/2 for downstream inhibition of Ras/MAPK pathway, although additional pathways may also exist (85). Interest in agonist targeting of CD200R in AD comes from data indicating expression on Th2 type cells involved in allergic responses. However, agonists LY345738 (ucenprubart) is currently in a Phase II study (NCT05911841) in AD after demonstrating safety in Phase I proof-of-concept study in healthy individuals. It is likely that CD200R agonist could be used for other indications based on expression patterns in disease, including treating cold urticaria by inhibiting mast cells (86).

### VSTM-1

V-set and transmembrane domain containing 1 (VSTM-1, SIRL-1) is a cell-surface inhibitory receptor highly expressed on granulocytes and subsets of monocytes (87). VSTM-1 inhibitory signaling is induced when the receptor binds to amphipathic alpha-helical damage-associated molecular pattern (DAMP) motifs on ligands such as cathelicidin and the S100 proteins (88, 89). VSTM-1 thus functions as a regulator of myeloid cell-driven inflammatory cascades. An agonist monoclonal antibody against VSTM-1 inhibited ERK1/2 signaling to suppress NETosis, production of reactive oxygen species (ROS), and cytokine release in inflammatory granulocytes *in vitro* and e*x vivo* (88, 90, 91). These preclinical data indicate that VSTM-1 may be a novel target for therapeutic intervention of granulocytic inflammatory disorders. While VSTM-1 is described here as a SuSt cell type therapeutic, it should be noted that in the context of cancer, where neutrophils and NETosis are associated with immune suppressive function and cancer progression, we speculate that agonism of VSTM-1 could act as a SuSu therapy to restore anti-tumor immunity.

### SIRPα

Antagonist blockers of the signal-regulatory protein alpha (SIRPα)-CD47 “don’t eat me” signal in phagocytic macrophages have long been pursued as anti-cancer therapeutics. SIRPα (CD172a) is an IIR shown to inhibit myeloid cell phagocytosis, migration, and activation (92). The development of SIRPα agonist IIRs has lagged significantly behind anti-cancer antagonists, but may be poised to make a comeback. A preclinical study by Xie et al. demonstrated increased SIRPα^+^ myeloid cells in inflamed tissue, and that an agonist mAb against SIRPα inhibited neutrophil and monocyte chemotaxis to ameliorate autoimmune joint inflammation or inflammatory colitis in mouse models of RA or inflammatory bowel disease (IBD), respectively (93).

### CMKLR1

CMKLR1 (Chemerin1/ChemR23) is a myeloid-expressed G protein-coupled receptor (GPCR) that binds lipidic resolvin E1 and chemerin to resolve the inflammatory plateau during activated immune responses (94). In multiple *in vitro* and *in vivo* models, an agonist antibody against CMKLR1 reduced tissue neutrophil accumulation, reprogrammed macrophage phenotype, and triggered resolution of chronic inflammation (95, 96).

Taken together, clinical and preclinical data for agonist antibodies targeting IIRs point to a surge of activity in such an approach and suggest a strong likelihood of approval of agonist IIR antibodies for inflammatory and autoimmune diseases. Moreover, the receptors discussed here comprise only a small number of the potential inhibitory receptors that have been identified, suggesting continued growth in this area for years to come.

## Small molecule IIR agonists in inflammation and autoimmune disease

Small molecule antagonism of ISR pathways (e.g. JAK-STAT pathways) has been well-documented for treating inflammation-associated, autoimmune, and fibrotic diseases (97, 98), and will not be discussed here. Likewise, small molecule antagonism of IIR pathways (e.g. PD-1, IDO) for treating cancer has been the basis of many current and developing therapeutics and can be reviewed elsewhere (99–101).

Studies investigating small molecule agonism of inhibitory pathways have lagged behind, likely because it is difficult to develop small molecule agonists for inhibitory receptor extracellular regions, and fewer specific, targetable intracellular inhibitory pathways have been identified in comparison to targetable stimulatory pathways. As such, there are currently no reported small molecule IIR agonists that are under development commercially, although this is likely to change, and may benefit from computational methodologies to predict IIR agonist molecules as SuSt-type therapeutics for inflammation-associated and autoimmune diseases.

Small molecules also have the potential to directly target IIRs on the cell surface and function as agonists, although most small molecule agonists currently target intracellular molecules (102). Indeed, extracellular region targeting may be a key for the advancement of small molecule agonists of IIRs. Functional specificity is likely to be enhanced by targeting extracellular regions of IIRs, since intracellular components are often involved in multiple cellular functions that could lead to broad, undesirable effects.

### VISTA/PD-1H

As mentioned earlier, Jun Liu’s group screened small molecules for identification of VISTA agonists by molecular docking, surface plasmon resonance, and cellular level experiments and discovered that VISTA agonists M351-0056 and imatinib, the latter an FDA approved tyrosine kinase inhibitor, alleviated lupus-like disease progression in chronic GvHD mice and in MRL/lpr mice by inhibiting activation of interferon I (IFN-I) and the noncanonical NF-κB pathway in monocytes (56, 103). Liu’s group went on to show that baloxavir marboxil, an antiviral drug for influenza, bound to VISTA and functions as an agonist to suppress lung inflammation in mouse asthma models (57). These studies were followed by a publication detailing discovery, synthesis and activity of small molecules targeting VISTA (104). These studies are significant in a) demonstrating small molecule agonism through extracellular binding of a cell surface receptor, b) supporting biologic-based agonism of the VISTA pathway, and finally c) for providing an additional avenue for targeting of VISTA during inflammation and autoimmunity, since generation of an anti-human agonist antibody has been challenging as demonstrated by the lack of such biologics under therapeutic development. A major caveat to these results is that imatinib and baloxavir marboxil are not specific for VISTA, although the above studies did demonstrate the requirement of VISTA for drug activity in the experimental settings that were tested.

### Intracellular phosphatases

Agonist targeting of IIR intracellular signaling components, such as phosphatases, as a means to induce immune inhibitory pathways, is not only challenging to study but has historically been unsuccessful therapeutically (105). However, small molecule targeting of phosphatases with antagonists or degraders continues to be of great interest. In fact, many of the phosphatases considered as targets for cancer with small molecule antagonists are the same phosphatases that would be ideal to target with agonists for inflammation and autoimmune indications. These include Src homology region-2 domain containing phosphatase-1 and 2 (SHP1 and SHP2, also called PTPN6 and PTPN11), protein tyrosine phosphatase 1B (PTB1B), and others reviewed here (105).

SC-43 and SC-78 are examples of orally available, small molecule agonists of SHP-1 (106). These agonists trigger conformational changes that inhibit STAT3 signaling. It is important to note that agonism of SHP-1 dephosphorylates JAK and STAT proteins, among others, and is thus very different from, for example, a direct STAT3 antagonist. Indeed, the nuance is that an SHP-1 agonist will regulate pathways in addition to STAT3 and thus have a broader effect, while at the same time only eliciting context dependent activity in the presence of stimulatory pathway activation. A study by Hong et al. indicated that targeting fibrosis-associated macrophages with SHP-1 agonists reduced idiopathic pulmonary fibrosis (107). It is likely that many other autoimmune and inflammation-associated diseases could benefit from SHP-1 agonists. It is possible that artificial intelligence initiatives may aid in the ability to develop potent, highly specific SHP-1 (or possibly SHP-2) agonist molecules.

Other immune inhibitory signaling molecules that can be targeted with small molecule agonists include SHIP1 (108) and PP2A (109). In addition, identification of specific cell surface receptors that preferentially induce these inhibitory pathways will allow for development of novel IIRs.

### Inhibitory G-α

GPCRs that signal through the inhibitory G-α (G-α) subunit to regulate the adenylyl cyclase (AC), cyclic-AMP (cAMP), and protein kinase A (PKA) pathways can be considered non-canonical inhibitors of immune function. For example, small molecule cannabinoid signaling through cannabinoid receptors, particularly CB2 expressed on populations of immune cells, is capable of suppressing immune-mediated inflammation (110).

### Immune Inhibitory Antibody Conjugates (IIACs)

It is also suggested here that, rather than immune stimulatory antibody conjugates, or ISACs, for the treatment of cancer, conjugating antibodies to small molecule agonist inhibitors, or immune inhibitory antibody conjugates (IIACs), should allow for an expanded set of targetable inhibitory pathways, and a broad new class of therapeutics. Moreover, utilizing small molecule agonists of phosphatases, G-alphai, SHIP1 and other pathways, combined with a cell surface antibody specific for immune cell subsets localized to inflammatory and autoimmune disease settings could confer highly specific activity to an inhibitory signaling component that may otherwise have broad activity, as may be the case with SHP-1 or G-alphai pathways.

Despite the challenges, the development of small molecule agonists of IIR pathways could be utilized in a variety of disease settings and would likely yield an improved safety profile in comparison to immune antagonists. Moreover, we predict that innovative advancements will take place in this area and look forward to the development of novel immune inhibitory antibody conjugate therapeutics.

## IIR agonists in cancer

In cancer, tremendous efforts have been made to understand and antagonize classic T cell checkpoint molecules such as PD-1 and CTLA-4 with the aim of untethering adaptive immune responses against cancerous cells (111, 112). More recently, the impact of non-T cell-centric IIRs, and the subsequent patient responses to immunotherapeutic intervention, have gained greater appreciation. Beyond Tregs, Myeloid-derived suppressor cells (MDSCs) are now recognized as major mediators of T cell suppression within the tumor microenvironment (TME). While the mechanisms that drive suppressive phenotypes are complex and beyond the scope of this review, IIR expression on both Tregs and MDSCs provide additional avenues for agonist therapeutics.

The concept of SuSu agonist therapeutics is particularly relevant in the context of the TME. Suppressor cells, including MSDC subpopulations, Tregs, and other suppressive populations that actively suppress an effective immune response could be targeted with IIR SuSu therapeutics to essentially suppress the suppressor cells, or more precisely, to suppress the suppressive functions of the suppressor cells. While there is little or no literature to highlight this concept in the context of agonist therapeutics, a few SuSu targets are described below that might effectively suppress suppressor cell function in the TME.

### VISTA

VISTA is one example of an IIR that is expressed on myeloid cells in solid tumor and AML (113, 114). Blockade of VISTA has demonstrated anti-leukemic effects, and since its initial characterization in 2011, VISTA has been targeted with blocking agents in multiple phase I and II clinical trials for the treatment of cancer (115, 116). However, agonist VISTA antibodies have not been tested in a cancer setting. Whether such antibodies would have activity in solid tumors remains to be determined. In particular, the balance of effects on VISTA agonist ability to suppress suppressive cell functionality, versus suppressing effector T cell functionality, would have to be considered in the same way that blockade of VISTA on effector T cells may also release suppressive cell functionality. The reason a VISTA agonist may be more appealing in this setting is that agonist signaling may only occur on cells expressing high levels of VISTA, where cross-linking is likely to occur more readily, thus leaving T cells that express lower levels of VISTA unaffected. In other words, it is possible that a VISTA agonist may be a more mechanistically specific, targeted therapeutic in comparison to a blocking VISTA mAb in the context of cancer.

### LAIR-1

Like VISTA, LAIR-1 was described above as a prime candidate for agonist targeting in inflammatory and autoimmune disease. However, LAIR-1 is also upregulated on several immune subsets in the TME of solid tumors (117–120), as well as on AML cells (67). Antagonist LAIR-1 therapies have been tested in clinical trials for the treatment of cancer, albeit with limited success to date (NCT05572684, NCT05215574).

Since LAIR-1 can be expressed on TAMs, MDSCs and other potentially suppressive subpopulations of immune cells, it is entirely possible, although untested, that agonist targeting of LAIR-1 could be a SuSu therapeutic, resulting in removal of suppressive pathways to strengthen anti-tumor immunity. Support for this notion comes from studies of LAIR-1 in heme cancers, as noted above. In leukemic cells LAIR-1 functions to suppress aberrant mTOR, NF-κB, and Akt self-renewal activity to promote apoptosis (67, 69, 121). It is also interesting that several studies have identified aberrant LAIR-1 expression on solid tumor cells, indicating that LAIR-1 may not be restricted to immune cells in the TME (118, 120, 122). It would be interesting to interrogate if agonist LAIR-1 antibodies could directly suppress tumor cell growth and potentially induce tumor cell death, as suggested by overexpressed of LAIR-1 on tumor cells signaling through the PI3K-AKT-mTOR axis (123).

### DR5

While DR5 (TNFRSF10B, TRAIL-R2) is not a canonical IIR, but rather induces apoptosis of cells through a death domain, some reports suggest the endogenous DR5 ligand, TRAIL, may also induce tumor progression, invasion, and metastasis (124). Some therapeutics targeting IIRs have sought to optimize clustering through multivalent modalities and FcR anchoring (reviewed in (125). A recent clinical example of a multivalent agonist mAb is the anti-DR5 antibody IGM-8444, which is an agonistic IgM antibody with 10 DR5 binding sites that results in inhibition of cells expressing DR5 (126). This is currently in a phase I trial for relapsed and/or refractory solid or hematologic cancers (NCT04553692).

Another interesting modality targeting DR5 is the tetravalent FAP-DR5 antibody RG7386. This construct utilizes anti-FAP Fab regions on one end of the molecule’s Fc domain and anti-DR5 domains on the other end, and hyper-clustering of DR5 on tumor cells can be induced by antibody bridging of DR5 on target cells with FAP expressed on cancer-associated fibroblasts (127). A Phase 1 dose escalation study in patients with advanced or metastatic solid tumors reported a favorable safety profile, 1 partial response, and 6 stable disease outcomes from 31 evaluated patients (NCT02558140).

## IIR agonist therapeutic opportunities and challenges

The fundamental difference in the IIR agonist approach compared to canonical antagonist strategies opens new areas of immunotherapeutic potential with both advantages and challenges. While it has typically been challenging to generate agonist mAbs, recent years have seen improvements in mAb generation technology in combination with a large expansion of companies utilizing artificial intelligence and machine learning strategies to optimize antibody constructs, as well as to identify, develop, or repurpose new small molecule agonist. It is therefore likely that this hurdle will be more readily overcome in the near future.

While an ideal agonist, whether biologic or small molecule, would not require molecular crosslinking or clustering, but rather binding to highly specific epitopes that optimally induce signaling when receptors are naturally clustered on cell surfaces, this may not be a therapeutically valid option in many cases where induced crosslinking or clustering is needed. Hopefully the concurrent advancement of biological understanding and engineering capabilities will allow for the development of novel, safe IIR agonist strategies that were previously unfeasible.

A potential benefit of agonist therapeutics is that the drug dosage required to reach efficacy is often much lower for agonist agents than for blocking agents. This is because, unlike blocking antagonists, agonist functionality rarely requires drug:receptor saturation (128–130). Indeed, drug response curves for many agonist molecules follow a bell curve model, with saturating concentrations actually decreasing activity, compared to the classic sigmoidal curve generated by antagonist drugs (131, 132). With this in mind, a drug may have a wider range of beneficial activity but may also present a challenge when attempting to determine the optimal dose based on current drug development strategies. In following, the therapeutic index for IIR agonists tends to be highly favorable, as significant efficacy may be observed at relatively low-dosage levels. Perhaps most importantly, because the mechanism of action (MOA) of IIR agonists is inhibition, and not activation, the risk of toxicity is highly mitigated (36). The counterpoint to the benefits of that IIR agonism that suppress specific immune function is that this may also leave patients more susceptible to pathogens or cancer, in some cases.

## Conclusion and future directions

Agonist therapeutics targeting IIRs is an exciting area of nascent therapeutic development with great promise against inflammation-associated and autoimmune diseases, and potentially in cancer based on emerging hypotheses and modalities. Advances in scientific understanding of immune modulatory receptor biology have increased the scope of therapeutic targets. Understanding not only biological mechanisms of action, but also therapeutic mechanisms of action, as well as contextual-based functional outcomes of agonist targeting of IIRs on a cellular level will open doors to optimal selection and use of IIR agonists for ideal indications. As new targets and therapeutics targeting IIRs emerge, careful preclinical and early clinical evaluation of biological effects remains paramount. The advancement of agonist targeting of IIRs in preclinical studies has led to clinical trials, but no currently approved drugs. Further research should be supported to develop IIR agonists for the clinic and patient benefit. As we enter an era of unprecedented technological advancement and the possibility for tailored drug design, it will be exciting to see how agonism of IIRs impacts the next generation of therapeutics.

## References

[1] Chen L Flies DB. Molecular mechanisms of T cell co-stimulation and co-inhibition. Nat Rev Immunol. (2013) 13:227–42. doi: 10.1038/nri3405 PMC3786574 23470321

[2] Su J Song Y Zhu Z Huang X Fan J Qiao J. Cell-cell communication: new insights and clinical implications. Signal Transduct Target Ther. (2024) 9:196. doi: 10.1038/s41392-024-01888-z 39107318 PMC11382761

[3] von Richthofen HJ Meyaard L. Sensing context: inhibitory receptors on non-hematopoietic cells. Eur J Immunol. (2023) 53:e2250306. doi: 10.1002/eji.202250306 36965113

[4] Ogawa S Abe R. Signal transduction via co-stimulatory and co-inhibitory receptors. Adv Exp Med Biol. (2019) 1189:85–133. doi: 10.1007/978-981-32-9717-3_4 31758532

[5] Billadeau DD Leibson PJ. Itams versus itims: striking a balance during cell regulation. J Clin Invest. (2002) 109:161–8. doi: 10.1172/JCI14843 PMC150845 11805126

[6] Worboys JD Davis DM. Do inhibitory receptors need to be proximal to stimulatory receptors to function? Genes Immun. (2024) 25:343–5. doi: 10.1038/s41435-023-00251-6 PMC11327099 38216665

[7] Rumpret M von Richthofen HJ Peperzak V Meyaard L. Inhibitory pattern recognition receptors. J Exp Med. (2022) 219(1):e20211463. doi: 10.1084/jem.20211463 34905019 PMC8674843

[8] Grebinoski S Vignali DA. Inhibitory receptor agonists: the future of autoimmune disease therapeutics? Curr Opin Immunol. (2020) 67:1–9. doi: 10.1016/j.coi.2020.06.001 32619929 PMC7744338

[9] Ribas A Wolchok JD. Cancer immunotherapy using checkpoint blockade. Science. (2018) 359:1350–5. doi: 10.1126/science.aar4060 PMC7391259 29567705

[10] He X Xu C. Immune checkpoint signaling and cancer immunotherapy. Cell Res. (2020) 30:660–9. doi: 10.1038/s41422-020-0343-4 PMC7395714 32467592

[11] Nakamura K Smyth MJ. Myeloid immunosuppression and immune checkpoints in the tumor microenvironment. Cell Mol Immunol. (2020) 17:1–12. doi: 10.1038/s41423-019-0306-1 31611651 PMC6952382

[12] Chan C Lustig M Baumann N Valerius T van Tetering G Leusen JHW. Targeting myeloid checkpoint molecules in combination with antibody therapy: A novel anti-cancer strategy with iga antibodies? Front Immunol. (2022) 13:932155. doi: 10.3389/fimmu.2022.932155 35865547 PMC9295600

[13] Liu X Hogg GD DeNardo DG. Rethinking immune checkpoint blockade: ‘Beyond the T cell’. J Immunother Cancer. (2021) 9(1):e001460. doi: 10.1136/jitc-2020-001460 33468555 PMC7817791

[14] Levi-Schaffer F Mandelboim O. Inhibitory and coactivating receptors recognising the same ligand: immune homeostasis exploited by pathogens and tumours. Trends Immunol. (2018) 39:112–22. doi: 10.1016/j.it.2017.10.001 PMC7106362 29066058

[15] Valls PO Esposito A. Signalling dynamics, cell decisions, and homeostatic control in health and disease. Curr Opin Cell Biol. (2022) 75:102066. doi: 10.1016/j.ceb.2022.01.011 35245783 PMC9097822

[16] Carvalheiro T Garcia S Pascoal Ramos MI Giovannone B Radstake T Marut W. Leukocyte associated immunoglobulin like receptor 1 regulation and function on monocytes and dendritic cells during inflammation. Front Immunol. (2020) 11:1793. doi: 10.3389/fimmu.2020.01793 32973751 PMC7466540

[17] Steevels TA Meyaard L. Immune inhibitory receptors: essential regulators of phagocyte function. Eur J Immunol. (2011) 41:575–87. doi: 10.1002/eji.201041179 21312193

[18] Paluch C Santos AM Anzilotti C Cornall RJ Davis SJ. Immune checkpoints as therapeutic targets in autoimmunity. Front Immunol. (2018) 9:2306. doi: 10.3389/fimmu.2018.02306 30349540 PMC6186808

[19] Sacdalan DB Lucero JA. The association between inflammation and immunosuppression: implications for ici biomarker development. Onco Targets Ther. (2021) 14:2053–64. doi: 10.2147/OTT.S278089 PMC7987319 33776452

[20] Baraibar I Melero I Ponz-Sarvise M Castanon E. Safety and tolerability of immune checkpoint inhibitors (Pd-1 and pd-L1) in cancer. Drug Saf. (2019) 42:281–94. doi: 10.1007/s40264-018-0774-8 30649742

[21] Meybodi SM Farasati Far B Pourmolaei A Baradarbarjastehbaf F Safaei M Mohammadkhani N. Immune checkpoint inhibitors promising role in cancer therapy: clinical evidence and immune-related adverse events. Med Oncol. (2023) 40:243. doi: 10.1007/s12032-023-02114-6 37453930

[22] Goulet DR Atkins WM. Considerations for the design of antibody-based therapeutics. J Pharm Sci. (2020) 109:74–103. doi: 10.1016/j.xphs.2019.05.031 31173761 PMC6891151

[23] Yakubu I Moinuddin I Brown A Sterling S Sinhmar P Kumar D. Costimulation blockade: the next generation. Curr Opin Organ Transplant. (2025) 30:96–102. doi: 10.1097/MOT.0000000000001206 39882641

[24] BaSudan AM. The role of immune checkpoint inhibitors in cancer therapy. Clin Pract. (2022) 13:22–40. doi: 10.3390/clinpract13010003 36648843 PMC9844484

[25] Adams AB Ford ML Larsen CP. Costimulation blockade in autoimmunity and transplantation: the cd28 pathway. J Immunol. (2016) 197:2045–50. doi: 10.4049/jimmunol.1601135 PMC5370073 27591335

[26] Ding M He Y Zhang S Guo W. Recent advances in costimulatory blockade to induce immune tolerance in liver transplantation. Front Immunol. (2021) 12:537079. doi: 10.3389/fimmu.2021.537079 33732228 PMC7959747

[27] McInnes IB Gravallese EM. Immune-mediated inflammatory disease therapeutics: past, present and future. Nat Rev Immunol. (2021) 21:680–6. doi: 10.1038/s41577-021-00603-1 PMC8436867 34518662

[28] Hunig T. The rise and fall of the cd28 superagonist tgn1412 and its return as tab08: A personal account. FEBS J. (2016) 283:3325–34. doi: 10.1111/febs.13754 27191544

[29] Yin N Li X Zhang X Xue S Cao Y Niedermann G. Development of pharmacological immunoregulatory anti-cancer therapeutics: current mechanistic studies and clinical opportunities. Signal Transduct Target Ther. (2024) 9:126. doi: 10.1038/s41392-024-01826-z 38773064 PMC11109181

[30] Ahmadzadeh M Johnson LA Heemskerk B Wunderlich JR Dudley ME White DE. Tumor antigen-specific cd8 T cells infiltrating the tumor express high levels of pd-1 and are functionally impaired. Blood. (2009) 114:1537–44. doi: 10.1182/blood-2008-12-195792 PMC2927090 19423728

[31] Muller D. Targeting co-stimulatory receptors of the tnf superfamily for cancer immunotherapy. BioDrugs. (2023) 37:21–33. doi: 10.1007/s40259-022-00573-3 36571696 PMC9836981

[32] Wang X Mathieu M Brezski RJ. Igg fc engineering to modulate antibody effector functions. Protein Cell. (2018) 9:63–73. doi: 10.1007/s13238-017-0473-8 28986820 PMC5777978

[33] Pauls SD Ray A Hou S Vaughan AT Cragg MS Marshall AJ. Fcgammariib-independent mechanisms controlling membrane localization of the inhibitory phosphatase ship in human B cells. J Immunol. (2016) 197:1587–96. doi: 10.4049/jimmunol.1600105 27456487

[34] Wang ZJ Thomson M. Localization of signaling receptors maximizes cellular information acquisition in spatially structured natural environments. Cell Syst. (2022) 13:530–46 e12. doi: 10.1016/j.cels.2022.05.004 35679857

[35] Toledo B Zhu Chen L Paniagua-Sancho M Marchal JA Peran M Giovannetti E. Deciphering the performance of macrophages in tumour microenvironment: A call for precision immunotherapy. J Hematol Oncol. (2024) 17:44. doi: 10.1186/s13045-024-01559-0 38863020 PMC11167803

[36] Zhai Y Moosavi R Chen M. Immune checkpoints, a novel class of therapeutic targets for autoimmune diseases. Front Immunol. (2021) 12:645699. doi: 10.3389/fimmu.2021.645699 33968036 PMC8097144

[37] Ma YV Sparkes A Romao E Saha S Gariepy J. Agonistic nanobodies and antibodies to human vista. MAbs. (2021) 13:2003281. doi: 10.1080/19420862.2021.2003281 34818120 PMC8632329

[38] Akinleye A Rasool Z. Immune checkpoint inhibitors of pd-L1 as cancer therapeutics. J Hematol Oncol. (2019) 12:92. doi: 10.1186/s13045-019-0779-5 31488176 PMC6729004

[39] Biemond M Vremec D Gray DH Hodgkin PD Heinzel S. Programmed death receptor 1 (Pd-1) ligand fc fusion proteins reduce T-cell proliferation *in vitro* independently of pd-1. Immunol Cell Biol. (2024) 102:117–30. doi: 10.1111/imcb.12714 PMC10952853 38069638

[40] Wang B Chen C Liu X Zhou S Xu T Wu M. The effect of combining pd-1 agonist and low-dose interleukin-2 on treating systemic lupus erythematosus. Front Immunol. (2023) 14:1111005. doi: 10.3389/fimmu.2023.1111005 36969198 PMC10030866

[41] Suzuki K Tajima M Tokumaru Y Oshiro Y Nagata S Kamada H. Anti-pd-1 antibodies recognizing the membrane-proximal region are pd-1 agonists that can down-regulate inflammatory diseases. Sci Immunol. (2023) 8:eadd4947. doi: 10.1126/sciimmunol.add4947 36638191

[42] Helou DG Quach C Fung M Painter JD Hurrell BP Eddie Loh YH. Human pd-1 agonist treatment alleviates neutrophilic asthma by reprogramming T cells. J Allergy Clin Immunol. (2023) 151:526–38 e8. doi: 10.1016/j.jaci.2022.07.022 35963455 PMC9905221

[43] Helou DG Shafiei-Jahani P Lo R Howard E Hurrell BP Galle-Treger L. Pd-1 pathway regulates ilc2 metabolism and pd-1 agonist treatment ameliorates airway hyperreactivity. Nat Commun. (2020) 11:3998. doi: 10.1038/s41467-020-17813-1 32778730 PMC7417739

[44] Curnock AP Bossi G Kumaran J Bawden LJ Figueiredo R Tawar R. Cell-targeted pd-1 agonists that mimic pd-L1 are potent T cell inhibitors. JCI Insight. (2021) 6(20):e152468. doi: 10.1172/jci.insight.152468 34491911 PMC8564903

[45] Bryan CM Rocklin GJ Bick MJ Ford A Majri-Morrison S Kroll AV. Computational design of a synthetic pd-1 agonist. Proc Natl Acad Sci U.S.A. (2021) 118:e2102164118. doi: 10.1073/pnas.2102164118 34272285 PMC8307378

[46] Flies DB Wang S Xu H Chen L. Cutting edge: A monoclonal antibody specific for the programmed death-1 homolog prevents graft-versus-host disease in mouse models. J Immunol (Baltimore Md: 1950). (2011) 187:1537–41. doi: 10.4049/jimmunol.1100660 PMC3150865 21768399

[47] Flies DB Han X Higuchi T Zheng L Sun J Ye JJ. Coinhibitory receptor pd-1h preferentially suppresses cd4+ T cell-mediated immunity. J Clin Invest. (2014) 124:1966–75. doi: 10.1172/JCI74589 PMC4001557 24743150

[48] Flies DB Higuchi T Chen L. Mechanistic assessment of pd-1h coinhibitory receptor-induced T cell tolerance to allogeneic antigens. J Immunol. (2015) 194:5294–304. doi: 10.4049/jimmunol.1402648 PMC4433880 25917101

[49] Borggrewe M Grit C Den Dunnen WFA Burm SM Bajramovic JJ Noelle RJ. Vista expression by microglia decreases during inflammation and is differentially regulated in cns diseases. Glia. (2018) 66:2645–58. doi: 10.1002/glia.23517 PMC6585704 30306644

[50] Wang L Rubinstein R Lines JL Wasiuk A Ahonen C Guo Y. Vista, a novel mouse ig superfamily ligand that negatively regulates T cell responses. J Exp Med. (2011) 208:577–92. doi: 10.1084/jem.20100619 PMC3058578 21383057

[51] Shekari N Shanehbandi D Kazemi T Zarredar H Baradaran B Jalali SA. Vista and its ligands: the next generation of promising therapeutic targets in immunotherapy. Cancer Cell Int. (2023) 23:265. doi: 10.1186/s12935-023-03116-0 37936192 PMC10631023

[52] Han X Vesely MD Yang W Sanmamed MF Badri T Alawa J. Pd-1h (Vista)-mediated suppression of autoimmunity in systemic and cutaneous lupus erythematosus. Sci Transl Med. (2019) 11:eaax1159. doi: 10.1126/scitranslmed.aax1159 31826980

[53] Liu H Li X Hu L Zhu M He B Luo L. A crucial role of the pd-1h coinhibitory receptor in suppressing experimental asthma. Cell Mol Immunol. (2018) 15:838–45. doi: 10.1038/cmi.2017.16 PMC6203798 28479600

[54] ElTanbouly MA Zhao Y Nowak E Li J Schaafsma E Le Mercier I. Vista is a checkpoint regulator for naive T cell quiescence and peripheral tolerance. Science. (2020) 367:eaay0524. doi: 10.1126/science.aay0524 31949051 PMC7391053

[55] Kim SH Adams TS Hu Q Shin HJ Chae G Lee SE. Vista (Pd-1h) is a crucial immune regulator to limit pulmonary fibrosis. Am J Respir Cell Mol Biol. (2023) 69:22–33. doi: 10.1165/rcmb.2022-0219OC 36450109 PMC10324045

[56] Yang L Zhang T Wang P Chen W Liu W He X. Imatinib and M351-0056 enhance the function of vista and ameliorate the development of sle via ifn-I and noncanonical nf-kappab pathway. Cell Biol Toxicol. (2023) 39:3287–304. doi: 10.1007/s10565-023-09833-6 37804401

[57] Di JW Wang YX Ma RX Luo ZJ Chen WT Liu WM. Repositioning baloxavir marboxil as vista agonist that ameliorates experimental asthma. Cell Biol Toxicol. (2024) 40:12. doi: 10.1007/s10565-024-09852-x 38340268 PMC10858940

[58] Tinoco R Otero DC Takahashi AA Bradley LM. Psgl-1: A new player in the immune checkpoint landscape. Trends Immunol. (2017) 38:323–35. doi: 10.1016/j.it.2017.02.002 PMC5411281 28262471

[59] Johnston RJ Su LJ Pinckney J Critton D Boyer E Krishnakumar A. Vista is an acidic ph-selective ligand for psgl-1. Nature. (2019) 574:565–70. doi: 10.1038/s41586-019-1674-5 31645726

[60] Huang CC Lu YF Wen SN Hsieh WC Lin YC Liu MR. A novel apoptosis-inducing anti-psgl-1 antibody for T cell-mediated diseases. Eur J Immunol. (2005) 35:2239–49. doi: 10.1002/eji.200525849 15948216

[61] Vanamee ES Faustman DL. The benefits of clustering in tnf receptor superfamily signaling. Front Immunol. (2023) 14:1225704. doi: 10.3389/fimmu.2023.1225704 37662920 PMC10469783

[62] Zhao P Xu Y Jiang L Fan X Li L Li X. A tetravalent trem2 agonistic antibody reduced amyloid pathology in a mouse model of alzheimer’s disease. Sci Transl Med. (2022) 14:eabq0095. doi: 10.1126/scitranslmed.abq0095 36070367

[63] Yang Y Yeh SH Madireddi S Matochko WL Gu C Pacheco Sanchez P. Tetravalent biepitopic targeting enables intrinsic antibody agonism of tumor necrosis factor receptor superfamily members. MAbs. (2019) 11:996–1011. doi: 10.1080/19420862.2019.1625662 31156033 PMC6748612

[64] Meyaard L. The inhibitory collagen receptor lair-1 (Cd305). J Leukoc Biol. (2008) 83:799–803. doi: 10.1189/jlb.0907609 18063695

[65] Jin J Wang Y Ma Q Wang N Guo W Jin B. Lair-1 activation inhibits inflammatory macrophage phenotype *in vitro* . Cell Immunol. (2018) 331:78–84. doi: 10.1016/j.cellimm.2018.05.011 29887420

[66] Guo N Zhang K Gao X Lv M Luan J Hu Z. Role and mechanism of lair-1 in the development of autoimmune diseases, tumors, and malaria: A review. Curr Res Transl Med. (2020) 68:119–24. doi: 10.1016/j.retram.2020.05.003 32690423

[67] Lovewell RR Hong J Kundu S Fielder CM Hu Q Kim KW. Lair-1 agonism as a therapy for acute myeloid leukemia. J Clin Invest. (2023) 133(22):e169519. doi: 10.1172/JCI169519 37966113 PMC10650974

[68] Van Laethem F Donaty L Tchernonog E Lacheretz-Szablewski V Russello J Buthiau D. Lair1, an itim-containing receptor involved in immune disorders and in hematological neoplasms. Int J Mol Sci. (2022) 23:16136. doi: 10.3390/ijms232416136 36555775 PMC9788452

[69] Poggi A Pellegatta F Leone BE Moretta L Zocchi MR. Engagement of the leukocyte-associated ig-like receptor-1 induces programmed cell death and prevents nf-kappab nuclear translocation in human myeloid leukemias. Eur J Immunol. (2000) 30:2751–8. doi: 10.1002/1521-4141(200010)30:10<2751::AID-IMMU2751>3.0.CO;2-L 11069054

[70] Meyaard L Adema GJ Chang C Woollatt E Sutherland GR Lanier LL. Lair-1, a novel inhibitory receptor expressed on human mononuclear leukocytes. Immunity. (1997) 7:283–90. doi: 10.1016/S1074-7613(00)80530-0 9285412

[71] van der Vuurst de Vries AR Clevers H Logtenberg T Meyaard L. Leukocyte-associated immunoglobulin-like receptor-1 (Lair-1) is differentially expressed during human B cell differentiation and inhibits B cell receptor-mediated signaling. Eur J Immunol. (1999) 29:3160–7. doi: 10.1002/(SICI)1521-4141(199910)29:10<3160::AID-IMMU3160>3.0.CO;2-S 10540327

[72] Rousseau A Parisi C Barlesi F. Anti-tigit therapies for solid tumors: A systematic review. ESMO Open. (2023) 8:101184. doi: 10.1016/j.esmoop.2023.101184 36933320 PMC10030909

[73] Joller N Hafler JP Brynedal B Kassam N Spoerl S Levin SD. Cutting edge: tigit has T cell-intrinsic inhibitory functions. J Immunol (Baltimore Md: 1950). (2011) 186:1338–42. doi: 10.4049/jimmunol.1003081 PMC3128994 21199897

[74] Dixon KO Schorer M Nevin J Etminan Y Amoozgar Z Kondo T. Functional anti-tigit antibodies regulate development of autoimmunity and antitumor immunity. J Immunol. (2018) 200:3000–7. doi: 10.4049/jimmunol.1700407 PMC5893394 29500245

[75] Lucca LE Axisa PP Singer ER Nolan NM Dominguez-Villar M Hafler DA. Tigit signaling restores suppressor function of th1 tregs. JCI Insight. (2019) 4:e124427. doi: 10.1172/jci.insight.124427 30728325 PMC6413794

[76] Kojima M Suzuki K Takeshita M Ohyagi M Iizuka M Yamane H. Anti-human-tigit agonistic antibody ameliorates autoimmune diseases by inhibiting tfh and tph cells and enhancing treg cells. Commun Biol. (2023) 6:500. doi: 10.1038/s42003-023-04874-3 37161050 PMC10170076

[77] Yu S Gu J Wang R Lee S Shan Y Wang J. Tigit reverses ifn-alpha-promoted th1-like tregs via in-sequence effects dependent on stat4. Arthritis Res Ther. (2023) 25:221. doi: 10.1186/s13075-023-03202-8 37978415 PMC10655484

[78] Hartigan CR Tong KP Liu D Laurie SJ Ford ML. Tigit agonism alleviates costimulation blockade-resistant rejection in a regulatory T cell-dependent manner. Am J Transplant. (2023) 23:180–9. doi: 10.1016/j.ajt.2022.12.011 PMC10062175 36695691

[79] Mao L Hou H Wu S Zhou Y Wang J Yu J. Tigit signalling pathway negatively regulates cd4(+) T-cell responses in systemic lupus erythematosus. Immunology. (2017) 151:280–90. doi: 10.1111/imm.12715 PMC5461105 28108989

[80] Guo Q Chen C Wu Z Zhang W Wang L Yu J. Engineered pd-1/tigit dual-activating cell-membrane nanoparticles with dexamethasone act synergistically to shape the effector T cell/treg balance and alleviate systemic lupus erythematosus. Biomaterials. (2022) 285:121517. doi: 10.1016/j.biomaterials.2022.121517 35504179

[81] Hasanzadeh S Farokh P Vazifeh F Hosseini GS Rezaei L Ghaedrahmati M. The efficacy and safety of relatlimab/nivolumab combination therapy in patients with advanced melanoma: A systematic review. Arch Dermatol Res. (2024) 317:65. doi: 10.1007/s00403-024-03579-9 39636334

[82] Angin M Brignone C Triebel F. A lag-3-specific agonist antibody for the treatment of T cell-induced autoimmune diseases. J Immunol. (2020) 204:810–8. doi: 10.4049/jimmunol.1900823 31907283

[83] Wojciechowicz K Spodzieja M Lisowska KA Wardowska A. The role of the btla-hvem complex in the pathogenesis of autoimmune diseases. Cell Immunol. (2022) 376:104532. doi: 10.1016/j.cellimm.2022.104532 35537322

[84] Vesely MD Lizzul PF Klekotka P Joller N Raff A. Immune checkpoint agonists for inflammatory skin diseases. J Invest Dermatol. (2025) 8. doi: 10.1016/j.jid.2025.01.008 PMC13281277 39927905

[85] Kotwica-Mojzych K Jodlowska-Jedrych B Mojzych M. Cd200:Cd200r interactions and their importance in immunoregulation. Int J Mol Sci. (2021) 22:1602. doi: 10.3390/ijms22041602 33562512 PMC7915401

[86] Metz M Kolkhir P Altrichter S Siebenhaar F Levi-Schaffer F Youngblood BA. Mast cell silencing: A novel therapeutic approach for urticaria and other mast cell-mediated diseases. Allergy. (2024) 79:37–51. doi: 10.1111/all.15850 37605867

[87] von Richthofen HJ Gollnast D van Capel TMM Giovannone B Westerlaken GHA Lutter L. Signal inhibitory receptor on leukocytes-1 is highly expressed on lung monocytes, but absent on mononuclear phagocytes in skin and colon. Cell Immunol. (2020) 357:104199. doi: 10.1016/j.cellimm.2020.104199 32942189

[88] Van Avondt K Fritsch-Stork R Derksen RH Meyaard L. Ligation of signal inhibitory receptor on leukocytes-1 suppresses the release of neutrophil extracellular traps in systemic lupus erythematosus. PloS One. (2013) 8:e78459. doi: 10.1371/journal.pone.0078459 24205237 PMC3799702

[89] Rumpret M von Richthofen HJ van der Linden M Westerlaken GHA Talavera Ormeno C Low TY. Recognition of S100 proteins by signal inhibitory receptor on leukocytes-1 negatively regulates human neutrophils. Eur J Immunol. (2021) 51:2210–7. doi: 10.1002/eji.202149278 PMC8457157 34145909

[90] Steevels TA Lebbink RJ Westerlaken GH Coffer PJ Meyaard L. Signal inhibitory receptor on leukocytes-1 is a novel functional inhibitory immune receptor expressed on human phagocytes. J Immunol. (2010) 184:4741–8. doi: 10.4049/jimmunol.0902039 20375307

[91] Steevels TA van Avondt K Westerlaken GH Stalpers F Walk J Bont L. Signal inhibitory receptor on leukocytes-1 (Sirl-1) negatively regulates the oxidative burst in human phagocytes. Eur J Immunol. (2013) 43:1297–308. doi: 10.1002/eji.201242916 23436183

[92] Barclay AN Van den Berg TK. The interaction between signal regulatory protein alpha (Sirpalpha) and cd47: structure, function, and therapeutic target. Annu Rev Immunol. (2014) 32:25–50. doi: 10.1146/annurev-immunol-032713-120142 24215318

[93] Xie MM Dai B Hackney JA Sun T Zhang J Jackman JK. An agonistic anti-signal regulatory protein alpha antibody for chronic inflammatory diseases. Cell Rep Med. (2023) 4:101130. doi: 10.1016/j.xcrm.2023.101130 37490914 PMC10439247

[94] Herova M Schmid M Gemperle C Hersberger M. Chemr23, the receptor for chemerin and resolvin E1, is expressed and functional on M1 but not on M2 macrophages. J Immunol. (2015) 194:2330–7. doi: 10.4049/jimmunol.1402166 25637017

[95] Lavy M Gauttier V Dumont A Chocteau F Deshayes S Fresquet J. Chemr23 activation reprograms macrophages toward a less inflammatory phenotype and dampens carcinoma progression. Front Immunol. (2023) 14:1196731. doi: 10.3389/fimmu.2023.1196731 37539056 PMC10396772

[96] Trilleaud C Gauttier V Biteau K Girault I Belarif L Mary C. Agonist anti-chemr23 mab reduces tissue neutrophil accumulation and triggers chronic inflammation resolution. Sci Adv. (2021) 7:eabd1453. doi: 10.1126/sciadv.abd1453 33811066 PMC11057782

[97] Jung SM Kim WU. Targeted immunotherapy for autoimmune disease. Immune Netw. (2022) 22:e9. doi: 10.4110/in.2022.22.e9 35291650 PMC8901705

[98] Hu X Li J Fu M Zhao X Wang W. The jak/stat signaling pathway: from bench to clinic. Signal Transduct Target Ther. (2021) 6:402. doi: 10.1038/s41392-021-00791-1 34824210 PMC8617206

[99] Sun H Li Y Zhang P Xing H Zhao S Song Y. Targeting toll-like receptor 7/8 for immunotherapy: recent advances and prospectives. biomark Res. (2022) 10:89. doi: 10.1186/s40364-022-00436-7 36476317 PMC9727882

[100] Wang Y Zhang S Li H Wang H Zhang T Hutchinson MR. Small-molecule modulators of toll-like receptors. Acc Chem Res. (2020) 53:1046–55. doi: 10.1021/acs.accounts.9b00631 32233400

[101] Wu Y Yang Z Cheng K Bi H Chen J. Small molecule-based immunomodulators for cancer therapy. Acta Pharm Sin B. (2022) 12:4287–308. doi: 10.1016/j.apsb.2022.11.007 PMC9764074 36562003

[102] Dhanak D Edwards JP Nguyen A Tummino PJ. Small-molecule targets in immuno-oncology. Cell Chem Biol. (2017) 24:1148–60. doi: 10.1016/j.chembiol.2017.08.019 28938090

[103] Hu X Qie C Jiang J Xie X Chen W Liu W. M351-0056 is a novel low mw compound modulating the actions of the immune-checkpoint protein vista. Br J Pharmacol. (2021) 178:1445–58. doi: 10.1111/bph.15357 PMC9328666 33450048

[104] Qi Z Cheng Y Wang K Cai S Ni X Wang T. Discovery, synthesis, and activity evaluation of novel small-molecule inhibitors targeting vista for cancer immunotherapy. J Med Chem. (2025) 68(5):5222–37. doi: 10.1021/acs.jmedchem.4c02031 40014385

[105] Kohn M. Turn and face the strange: A new view on phosphatases. ACS Cent Sci. (2020) 6:467–77. doi: 10.1021/acscentsci.9b00909 PMC7181316 32341996

[106] Chung SY Chen YH Lin PR Chao TC Su JC Shiau CW. Two novel shp-1 agonists, sc-43 and sc-78, are more potent than regorafenib in suppressing the *in vitro* stemness of human colorectal cancer cells. Cell Death Discovery. (2018) 4:25. doi: 10.1038/s41420-018-0084-z PMC6089896 30109144

[107] Hong SY Lu YT Chen SY Hsu CF Lu YC Wang CY. Targeting pathogenic macrophages by the application of shp-1 agonists reduces inflammation and alleviates pulmonary fibrosis. Cell Death Dis. (2023) 14:352. doi: 10.1038/s41419-023-05876-z 37291088 PMC10249559

[108] Ong CJ Ming-Lum A Nodwell M Ghanipour A Yang L Williams DE. Small-molecule agonists of ship1 inhibit the phosphoinositide 3-kinase pathway in hematopoietic cells. Blood. (2007) 110:1942–9. doi: 10.1182/blood-2007-03-079699 17502453

[109] McClinch K Avelar RA Callejas D Izadmehr S Wiredja D Perl A. Small-molecule activators of protein phosphatase 2a for the treatment of castration-resistant prostate cancer. Cancer Res. (2018) 78:2065–80. doi: 10.1158/0008-5472.CAN-17-0123 PMC5899650 29358171

[110] Mackie K. Cannabinoid receptors: where they are and what they do. J Neuroendocrinol. (2008) 20 Suppl 1:10–4. doi: 10.1111/j.1365-2826.2008.01671.x 18426493

[111] Simon S Labarriere N. Pd-1 expression on tumor-specific T cells: friend or foe for immunotherapy? Oncoimmunology. (2017) 7:e1364828. doi: 10.1080/2162402X.2017.1364828 29296515 PMC5739549

[112] Lee JB Kim HR Ha SJ. Immune checkpoint inhibitors in 10 years: contribution of basic research and clinical application in cancer immunotherapy. Immune Netw. (2022) 22:e2. doi: 10.4110/in.2022.22.e2 35291660 PMC8901707

[113] Martin AS Molloy M Ugolkov A von Roemeling RW Noelle RJ Lewis LD. Vista expression and patient selection for immune-based anticancer therapy. Front Immunol. (2023) 14:1086102. doi: 10.3389/fimmu.2023.1086102 36891296 PMC9986543

[114] Zhang RJ Kim TK. Vista-mediated immune evasion in cancer. Exp Mol Med. (2024) 56:2348–56. doi: 10.1038/s12276-024-01336-6 PMC11612309 39482534

[115] Kim TK Han X Hu Q Vandsemb EN Fielder CM Hong J. Pd-1h/vista mediates immune evasion in acute myeloid leukemia. J Clin Invest. (2024) 134:e164325. doi: 10.1172/JCI164325 38060328 PMC10836799

[116] Kamali AN Bautista JM Eisenhut M Hamedifar H. Immune checkpoints and cancer immunotherapies: insights into newly potential receptors and ligands. Ther Adv Vaccines Immunother. (2023) 11:25151355231192043. doi: 10.1177/25151355231192043 37662491 PMC10469281

[117] Flies DB Higuchi T Harris JC Jha V Gimotty PA Adams SF. Immune checkpoint blockade reveals the stimulatory capacity of tumor-associated cd103(+) dendritic cells in late-stage ovarian cancer. Oncoimmunology. (2016) 5:e1185583. doi: 10.1080/2162402X.2016.1185583 27622059 PMC5007952

[118] Cao Q Fu A Yang S He X Wang Y Zhang X. Leukocyte-associated immunoglobulin-like receptor-1 expressed in epithelial ovarian cancer cells and involved in cell proliferation and invasion. Biochem Biophys Res Commun. (2015) 458:399–404. doi: 10.1016/j.bbrc.2015.01.127 25660999

[119] Jingushi K Uemura M Nakano K Hayashi Y Wang C Ishizuya Y. Leukocyte−Associated immunoglobulin−Like receptor 1 promotes tumorigenesis in rcc. Oncol Rep. (2019) 41:1293–303. doi: 10.3892/or.2018.6875 30483814

[120] Wang Y Zhang X Miao F Cao Y Xue J Cao Q. Clinical significance of leukocyte-associated immunoglobulin-like receptor-1 expression in human cervical cancer. Exp Ther Med. (2016) 12:3699–705. doi: 10.3892/etm.2016.3842 PMC5228450 28105100

[121] Zocchi MR Pellegatta F Pierri I Gobbi M Poggi A. Leukocyte-associated ig-like receptor-1 prevents granulocyte-monocyte colony stimulating factor-dependent proliferation and akt1/pkb alpha activation in primary acute myeloid leukemia cells. Eur J Immunol. (2001) 31:3667–75. doi: 10.1002/1521-4141(200112)31:12<3667::aid-immu3667>3.0.co;2-g 11745387

[122] Aung TN Gavrielatou N Vathiotis IA Fernandez AI Shafi S Yaghoobi V. Quantitative, spatially defined expression of leukocyte-associated immunoglobulin-like receptor in non-small cell lung cancer. Cancer Res Commun. (2023) 3:471–82. doi: 10.1158/2767-9764.CRC-22-0334 PMC10029762 36960400

[123] Liu Y Ma L Shangguan F Zhao X Wang W Gao Z. Lair-1 suppresses cell growth of ovarian cancer cell via the pi3k-akt-mtor pathway. Aging (Albany NY). (2020) 12:16142–54. doi: 10.18632/aging.103589 PMC7485720 32628130

[124] Guerrache A Micheau O. Tnf-related apoptosis-inducing ligand: non-apoptotic signalling. Cells. (2024) 13:521. doi: 10.3390/cells13060521 38534365 PMC10968836

[125] Jhajj HS Lwo TS Yao EL Tessier PM. Unlocking the potential of agonist antibodies for treating cancer using antibody engineering. Trends Mol Med. (2023) 29:48–60. doi: 10.1016/j.molmed.2022.09.012 36344331 PMC9742327

[126] Wang BT Kothambawala T Wang L Matthew TJ Calhoun SE Saini AK. Multimeric anti-dr5 igm agonist antibody igm-8444 is a potent inducer of cancer cell apoptosis and synergizes with chemotherapy and bcl-2 inhibitor abt-199. Mol Cancer Ther. (2021) 20:2483–94. doi: 10.1158/1535-7163.MCT-20-1132 PMC9398157 34711645

[127] Brunker P Wartha K Friess T Grau-Richards S Waldhauer I Koller CF. Rg7386, a novel tetravalent fap-dr5 antibody, effectively triggers fap-dependent, avidity-driven dr5 hyperclustering and tumor cell apoptosis. Mol Cancer Ther. (2016) 15:946–57. doi: 10.1158/1535-7163.MCT-15-0647 27037412

[128] Wang R Gao C Raymond M Dito G Kabbabe D Shao X. An integrative approach to inform optimal administration of ox40 agonist antibodies in patients with advanced solid tumors. Clin Cancer Res. (2019) 25:6709–20. doi: 10.1158/1078-0432.CCR-19-0526 31573956

[129] Waibler Z Sender LY Kamp C Muller-Berghaus J Liedert B Schneider CK. Toward experimental assessment of receptor occupancy: tgn1412 revisited. J Allergy Clin Immunol. (2008) 122:890–2. doi: 10.1016/j.jaci.2008.07.049 18805577

[130] Stebbings R Findlay L Edwards C Eastwood D Bird C North D. Cytokine storm” in the phase I trial of monoclonal antibody tgn1412: better understanding the causes to improve preclinical testing of immunotherapeutics. J Immunol. (2007) 179:3325–31. doi: 10.4049/jimmunol.179.5.3325 17709549

[131] Nordstrom JL Gorlatov S Zhang W Yang Y Huang L Burke S. Anti-tumor activity and toxicokinetics analysis of mgah22, an anti-her2 monoclonal antibody with enhanced fcgamma receptor binding properties. Breast Cancer Res. (2011) 13:R123. doi: 10.1186/bcr3069 22129105 PMC3326565

[132] Brahmer JR Drake CG Wollner I Powderly JD Picus J Sharfman WH. Phase I study of single-agent anti-programmed death-1 (Mdx-1106) in refractory solid tumors: safety, clinical activity, pharmacodynamics, and immunologic correlates. J Clin Oncol: Off J Am Soc Clin Oncol. (2010) 28:3167–75. doi: 10.1200/JCO.2009.26.7609 PMC4834717 20516446

